# Computational Study of Symmetric Methylation on Histone Arginine Catalyzed by Protein Arginine Methyltransferase PRMT5 through QM/MM MD and Free Energy Simulations

**DOI:** 10.3390/molecules200610032

**Published:** 2015-05-29

**Authors:** Yufei Yue, Yuzhuo Chu, Hong Guo

**Affiliations:** 1 Department of Biochemistry and Cellular and Molecular Biology, University of Tennessee, Knoxville, TN 37996, USA; E-Mail: yyue@vols.utk.edu; 2 School of Life Science and Biotechnology, Dalian University of Technology, Dalian 116024, China; E-Mail: yzchu@dlut.edu.cn; 3 UT/ORNL Center for Molecular Biophysics, Oak Ridge National Laboratory, Oak Ridge, TN 37830, USA

**Keywords:** protein arginine methyltransferase (PRMT), symmetric dimethylarginine (SDMA), asymmetric dimethylarginine (ADMA)

## Abstract

Protein arginine methyltransferases (PRMTs) catalyze the transfer of the methyl group from *S*-adenosyl-l-methionine (AdoMet) to arginine residues. There are three types of PRMTs (I, II and III) that produce different methylation products, including asymmetric dimethylarginine (ADMA), symmetric dimethylarginine (SDMA) and monomethylarginine (MMA). Since these different methylations can lead to different biological consequences, understanding the origin of product specificity of PRMTs is of considerable interest. In this article, the quantum mechanical/molecular mechanical (QM/MM) molecular dynamics (MD) and free energy simulations are performed to study SDMA catalyzed by the Type II PRMT5 on the basis of experimental observation that the dimethylated product is generated through a distributive fashion. The simulations have identified some important interactions and proton transfers during the catalysis. Similar to the cases involving Type I PRMTs, a conserved Glu residue (Glu435) in PRMT5 is suggested to function as general base catalyst based on the result of the simulations. Moreover, our results show that PRMT5 has an energetic preference for the first methylation on N_η1_ followed by the second methylation on a different ω-guanidino nitrogen of arginine (N_η2_).The first and second methyl transfers are estimated to have free energy barriers of 19–20 and 18–19 kcal/mol respectively. The computer simulations suggest a distinctive catalytic mechanism of symmetric dimethylation that seems to be different from asymmetric dimethylation.

## 1. Introduction

**Scheme 1:**
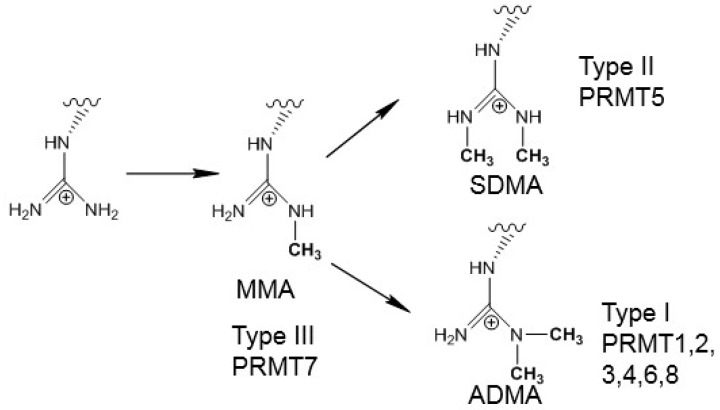
Methylation of arginine by different types of PRMTs. Type I PRMT can produce both monomethylarginine (MMA) and asymmetric dimethylarginine (ADMA). Type II PRMT can produce both MMA and symmetric dimethylarginine (SDMA). Type III PRMT can only produce MMA.

Posttranslational methylation of histone proteins on their arginine residues is an epigenetic mark that plays a vital role in cell function and is related with cell disorders and diseases [1]. Protein arginine methyltransferases (PRMTs) catalyze the transfer of methyl group(s) from S-adenosyl-l-methionine (AdoMet) to the guanidine nitrogen of arginine residue, resulting in the reaction products containing methylarginine along with S-adenosylhomocysteine (SAH) [2]. In addition to the methylation of histone proteins, PRMTs could also modify a variety of other proteins [3]. Depending on types of PRMTs (I, II or III), the methylation products may contain asymmetric dimethylarginine (ADMA), symmetric dimethylarginine (SDMA) or monomethylarginine (MMA) (shown in Scheme 1) [1].

The Type II PRMT5 to be investigated in this work catalyzes the transfer of methyl groups to the two different ω-guanidino nitrogen atoms on the arginine residue of the target protein, producing the ω-N^G^, N′^G^ symmetrically dimethylated arginine (SDMA) [4]. PRMT5 has a variety of substrates that include histones, transcription factors, splicesomal proteins and piRNA biogenesis related proteins, and this enzyme functions in both nucleus and cytoplasm [3]. SDMA may profoundly impact many biological processes including epigenetic control of gene expression [5], circadian rhythms [6,7], splicing regulation [8,9], germ cell development and pluripotency [10,11], and DNA damage response [12,13]. The Type II PRMT5, however, often share the common recognition sequence with the Type I PRMTs which add two methyl groups to the same ω-guanidino nitrogen atom (ADMA) [14]. Thus, the same target arginine may be either symmetrically or asymmetrically dimethylated. As such, SDMA and ADMA are isomeric protein posttranslational modifications with distinct and sometimes reversible biological effects [14]. One example is the methylation of arginine 3 on histone H4 (H4R3): symmetric dimethylation of H4R3 could repress gene expression [15,16], while asymmetric dimethylation of H4R3 is correlated with gene activation [17,18]. Recent studies have focused on understanding the enzymatic mechanisms that differentiate the two chemically isomeric but functionally antagonistic posttranslational modifications [14].

The crystal structures have been determined for several PRMTs, including PRMT1 [19], PRMT3 [20], PRMT6 [21], PRMT10 [22] of Type I, PRMT5 [23] of Type II and PRMT7 [24] of Type III. Computer simulations have been applied to investigate the catalytic mechanism of PRMT1 [25] and PRMT3 [26], and some important questions concerning the product specificity of Type I ADMA have been addressed. The computational approaches used in these earlier studies include molecular dynamics and free energy simulations (potential of mean force) with the hybrid quantum mechanical and molecular mechanical (QM/MM) potential that seem to be suitable to investigate the enzyme-catalyzed methyl transfer process and have also been widely used for some other methyltransferases [26,27,28,29,30,31,32].

In this study, the methylation reaction catalyzed by Type II PRMT5 is investigated by use of QM/MM MD and free energy simulations on the basis of experimental observation that the dimethylated product is generated through a distributive fashion. The simulations have identified some important interactions and proton transfers involving the active site residues. Similar to the cases of Type I PRMT1 [25] and PRMT3 [26], a homologous Glu residue (Glu435) in PRMT5 seems to function as a general base catalyst during the catalysis, and the corresponding proton transfer is found to be somehow concerted with the methyl transfer process. However, unlike Type I PRMTs which energetically favors a single ω-guanidino nitrogen (N_η2_) of arginine as the target for the both 1st and 2nd methylations [25,26], PRMT5 is found to have an energetic preference of targeting N_η1_ for the first methylation and then targeting a different ω-guanidino nitrogen (N_η2_) for the second methylation. The first and second methyl transfers are estimated to have free energy barriers of 19–20 and 18–19 kcal/mol, respectively, from the simulations. These results are consistent with the existing experimental data [23]. Our computational study provides a better understanding of the symmetric di-methylation mechanism that is different from the asymmetric di-methylation.

## 2. Results and Discussion

### 2.1. Comparison of the Active Site Structures of Type II PRMT5 and Type I PRMT3

**Figure 1:**
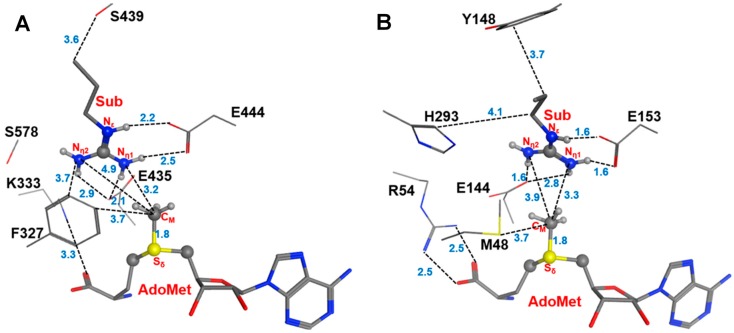
Comparison of the active sites of the crystal structures for Type-II PRMT5 (PDB ID: 4QGB) and Type-I PRMT3 (PDB ID:1F3L). (**A**) The active site of PRMT5; (**B**).The active site of PRMT3. The two glutamate residues, E435 (E144) and E444 (E153) in PRMT5 (PRMT3), are conserved in the both types of PRMTs. F327, K333, S578 and S439 in PRMT5 are the conserved residues among Type-II PRMTs. The corresponding residues in Type-I PRMT3 are M48, R54, H293 and Y148, respectively. The substrate arginine is labeled as Sub. Some distances are shown in blue with the unit of angstrom.

The two invariant glutamate residues from the “double-E” loop found in all PRMTs [33] are structurally conserved in both Type I PRMT3 and Type II PRMT5 active sites (E435 and E444 in Figure 1A; E144 and E153 in Figure 1B). As is shown in Figure 1, the carboxylate side chain of E444 in PRMT5 (E153 in PRMT3) forms a stable salt bridge with the N_ε_ and N_η1_ atoms of the substrate arginine. The side chain of E435 in PRMT5 (E144 PRMT3) interacts with N_η1_ and N_η2_ of the substrate. These two Glu residues are required for the enzymatic activities; the mutation of either of them could greatly decrease the enzymatic activity [14].

Previous computational studies have identified E144 as the general base to accept proton from the arginine during the methyl transfer catalyzed by Type I PRMTs [25,26]. One interesting questions is whether the corresponding E435 from PRMT5 would play a similar role during the catalysis. Four other residues that are conserved in the active site of PRMT5 are F327, K333, S578 and S439; the corresponding residues in PRMT3 are M48, R54, Y148 and H293, respectively. Although the mutation of S439 and S578 of PRMT5 diminishes the enzymatic activity significantly [14], the exact role of these residues is not clear; both S439 and S578 seem to be far away from the methyl donating AdoMet (Figure 1A). Interestingly, the F327M mutant of PRMT5 could produce both ADMA and SDMA [14], indicating that F327 may occupy a key position in PRMT5 and its properties may be important in determining the product specificity. Moreover, K333 is in the vicinity of F327 and could form hydrogen bonds with both the carboxylate group of AdoMet and E435.

The previous computational studies have shown that for PRMT1 and PRMT3 the 1st and 2nd methyl transfers would be energetically more favorable with relatively lower barriers if N_η2_ (see Figure 1B) is the methyl acceptor in each of the cases [25,26]. This is consistent with the fact that they are both Type I PRMTs and the methylation products contain asymmetric dimethylarginine (ADMA). In the active site of the PRMT5 structure (Figure 1A), N_η1_ appears to be in a much better position for acting as the methyl acceptor compared to N_η2_; this is in contrast with the case of PRMT3 where N_η2_ seems to be in a better position for accepting the methyl group [26]. Indeed, the distance between N_η1_ and C_M_ is 3.2 Å in the active site of PRMT5 as compared to 4.9 Å between N_η2_ and C_M_.

### 2.2. The First Methylation Catalyzed by PRMT5

**Figure 2:**
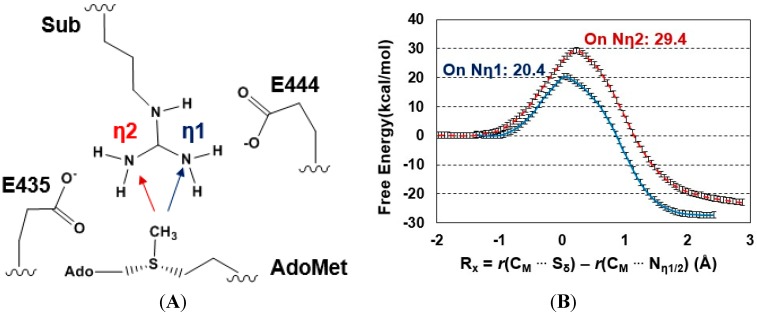
(**A**) Two possible sites for the 1st methylation. The substrate arginine is labeled as Sub; (**B**) Free energy profiles of the 1st methylation on N_η1_ and N_η2_. To N_η1_: blue line with a free energy barrier of 20.4 kcal/mol; to N_η2_: red dotted line with a free energy barrier of 29.4 kcal/mol. Error bars are shown on the free energy profile (within ± 0.12 kcal/mol).

The acceptor site for the first methylation process catalyzed by PRMT5 is studied by comparing the energetics of the methyl addition to N_η1_ and N_η2_, respectively, on the arginine via QM/MM MD and free energy simulations. As shown in Figure 2B, the free energy barrier for the methyl transfer to N_η1_ is 20.4 kcal/mol, which is 9 kcal/mol lower than that for the methyl transfer to N_η2_. This suggests that the 1st methyl group is likely to be transferred to the N_η1_ atom, as the corresponding process is energetically more favorable. This is in contrast with the 1st methylation catalyzed by PRMT3 which favors N_η2_ as the methyl acceptor [26].

**Figure 3:**
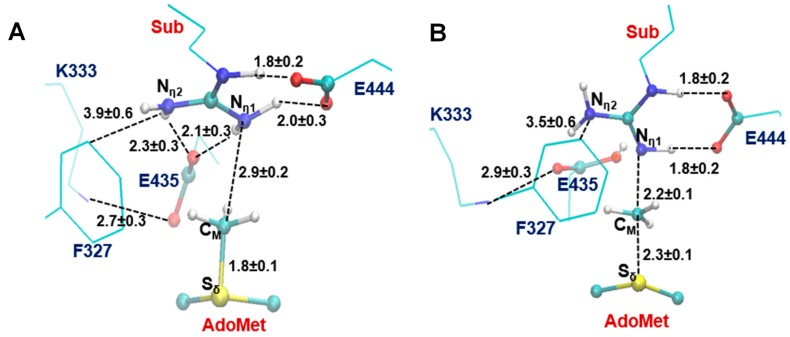
The average active-site structures obtained from the MD and free energy simulations for the 1st methylation on N_η1_. (**A**) Reactant complex; (**B**) Near the transition state. Some average distances in active sites are shown in angstrom. Note that E435 works as the proton acceptor based on the simulations.

**Figure 4:**
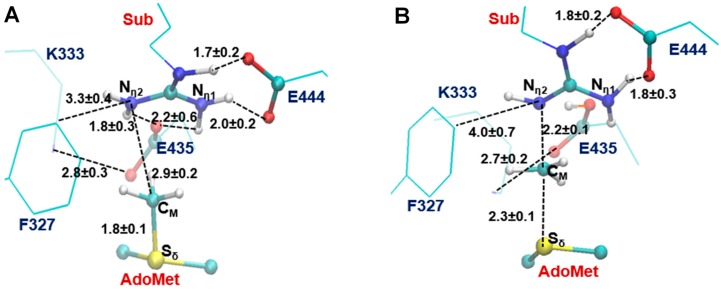
The average active-site structure obtained from the MD and free energy simulations for the 1st methylation on N_η2_. (**A**) Reactant complex; (**B**) Near the transition state.

The average active-site structures of the reactant complex and near transition state for the 1st methyl transfer are shown in Figure 3 and Figure 4 for the methyl transfer to N_η1_ and N_η2_, respectively. The interactions obtained from the simulations for the reactant complex generally resemble the interactions observed in the crystal structure. This seems to indicate that the conformation of the active site in the crystal structure is well maintained after initial 1.5 ns MD simulations. In the reactant complex for the methyl transfer to N_η1_ (Figure 3A), N_η1_ is well aligned with the methyl group of AdoMet, and this good alignment presumably leads to a relatively low barrier for the methyl transfer. The arginine on the substrate appears to be well stabilized through the salt bridge with E444 as well as hydrogen bonding interaction with E435. K333 may also help to adjust the orientation of E435 through a hydrogen bonding interaction. F327 seems to be involved in the π-cation interaction with the arginine in the reactant state. Moreover, as mentioned earlier the bulk size of this residue may prevent the formation of a good alignment between the transferable methyl group and N_η2_ and interfere with the methyl transfer to N_η2_. Indeed, the structures in Figure 1A shows that the distance between the transferable methyl group (C_M_) and N_η2_ can be as much as 4.9 Å in the reactant complex. By contrast, the corresponding distance in PRMT3, which has M48 at the location rather than a Phe residue, is much smaller (e.g., 3.9 Å in Figure 1B). It is of interest to note from Figure 4B that the distance between F327 and the arginine increases significantly near the transition state during the methyl transfer to N_η2_.

**Figure 5:**
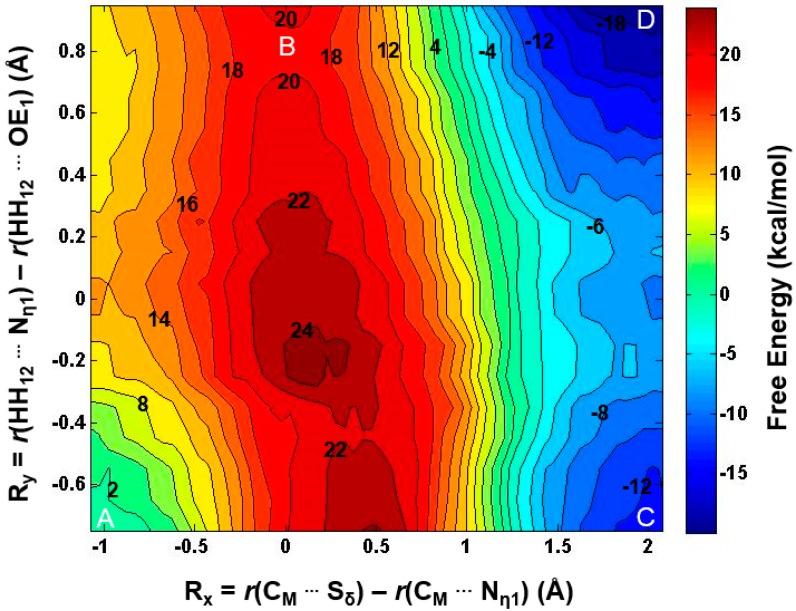
2D free-energy contour map for the 1st methylation on N_η1_. R_x_: the reaction coordinate of the methyl transfer; R_y_: the reaction coordinate for the proton transfer. Points A, B, and D designate the reactant, near transition state, and product complexes, respectively. Some contour lines are shown with respective energy values. The energy barrier is estimated to be around 19–20 kcal/mol.

The 1D free energy simulations only used the reaction coordinate for the methyl transfer. Nevertheless, the proton transfer occurred near the transition state of methylation (Figure 3B and Figure 4B). To better understand the relationship between the methyl transfer and the proton transfer process, the 2D free energy simulations were performed (Figure 5). As is shown in Figure 5, the reaction path goes from point A (designated as the reactant complex) to point B (the transition state), and finally reaches point D as the product complex. Before reaching the transition state at point B, the proton has been basically transferred from N_η1_ of the substrate arginine to the carboxyl oxygen of E435. And the free energy barrier is estimated to be 19–20 kcal/mol from the 2D free energy simulations, which is almost the same as that obtained from the 1D free energy simulations (20.4 kcal/mol). Moreover, point D in Figure 5 is about 6 kcal/mol lower than point C, suggesting that the deprotonated product is more stable in the active site than the protonated product and that the proton transfer is coupled with the methyl transfer during methylation.

### 2.3. The Second Methylation Catalyzed by PRMT5

With the first methyl group being transferred already, the 2nd methylation reaction can now be examined to determine the mechanism for the formation of the symmetrically dimethylated product. Previous experimental study on *Caenorhabditis elegans* PRMT5 (cPRMT5) has shown that the dimethylated product is generated through a distributive fashion [4] in which the peptide is released prior to rebinding to facilitate a second round of methylation. The distributive mechanism was further confirmed from the study of the human hPRMT5•MEP50 complex [34]. This is in contrast with the cases of some protein lysine methyltransferases for which the multiple rounds of methylation are believed to proceed processively without the release of the intermediates from the active sites. The kinetic data (*K_m_* and *k_cat_*) for cPRMT5, hPRMT5 and hPRMT5•MEP50 complex have also been obtained for a variety of un-methylated and mono-methylated substrates. For the hPRMT5•MEP50 complex for which the current investigation is based on, the differences in *K_m_* and *k_cat_* for the first and second methyl transfers are rather small and are beyond the accuracy of most of quantum mechanical approaches used in QM/MM studies. Although several experimental structures for PRMTs are available, the structure for the reactant complex of the second methyl transfer has not been determined. In our earlier investigations of protein lysine methyltransferases [27,28,29,30], we developed an approach based on the free energy simulations of the methyl transfer processes that allows us to determine whether or not the enzymes would be able to add methyl groups. This approach has been successfully applied to study the product specificity for a number of protein lysine methyltransferases, and its usefulness has also been confirmed on some other methyltransferases [26,31,32], including PRMT1 [26]. This approach is especially suitable for the current investigation because the *K_m_* values for the first and second methyl transfers are rather similar (see above), and the hypothetical non-existent methyl transfers can be presumably eliminated using this approach in the similar manner as the product specificity is determined [26,27,28,29,30]. In Table 1, the three different configurations for the mono-methyl arginine without destroying the salt bridge with E444 are given. The nitrogen atom already connecting to the methyl group is designated as N_η1_ and that without the methyl group (*i.e.*, NH_2_) designated as N_η2_. As is demonstrated in Table 1, for each configuration there are two possible di-methylation products (SDMA and ADMA). The QM/MM free energy simulations have been performed for each of the six methylation processes. Table 1(I) shows that the free energy barriers were calculated to be 32.3 and 36 kcal/mol for the 2nd methyl transfer to the N_η1_ and N_η2_ atoms, respectively. Since either of these energy barriers seems to be too high, the 2nd methylation may not start from the configuration in Table 1(I). The similar argument can be made for the configuration in Table 1(III).

**Table 1:** Free energy barriers for the 2nd methylation on either the mono-methylated nitrogen atom (designated as N_η1_) or un-methylated nitrogen atom (designated as N_η2_) of the mono-methylated arginine (MMA) with three possible configurations for the reactant complex without destroying the salt bridge involving E444. The only difference between Configurations II and III is whether the methyl group is pointing to the AdoMet side (III) or away from the AdoMet side (II). The conformation in (II) has the lowest energy barrier of 20.1 kcal/mol when the 2nd methylation occurs to N_η2_ which will eventually produce the symmetrically dimethylated arginine (SDMA). Error bars are also determined, similar to those given in Figure 2 (within ± 0.2 kcal/mol).

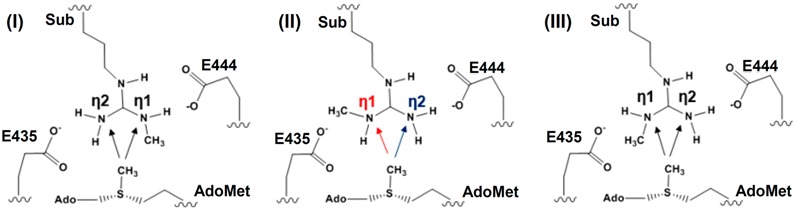					
To N_η1_	To N_η2_	To N_η1_	To N_η2_	To N_η1_	To N_η2_
32.3 kcal/mol	36 kcal/mol	31.3 kcal/mol	20.1 kcal/mol	43.9 kcal/mol	32.5 kcal/mol

Table 1(II) shows that the second methyl transfer has the lowest free energy barrier (20.1 kcal/mol) if it is transferred to N_η2_ (*i.e.*, the SDMA product), while the free energy barrier for the second methyl transfer to N_η1_ (*i.e.*, the ADMA product) based on the configuration in Table 1(II) is 11.2 kcal/mol higher.

**Figure 6:**
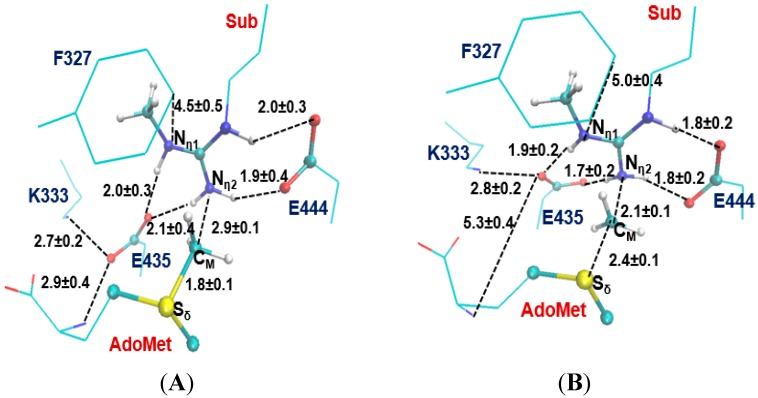
Results from the simulations for the 2nd methylation on N_η2_. (**A**) Active-site structure of the reactant complex; (**B**) Active-site structure near the transition state.

**Figure 7:**
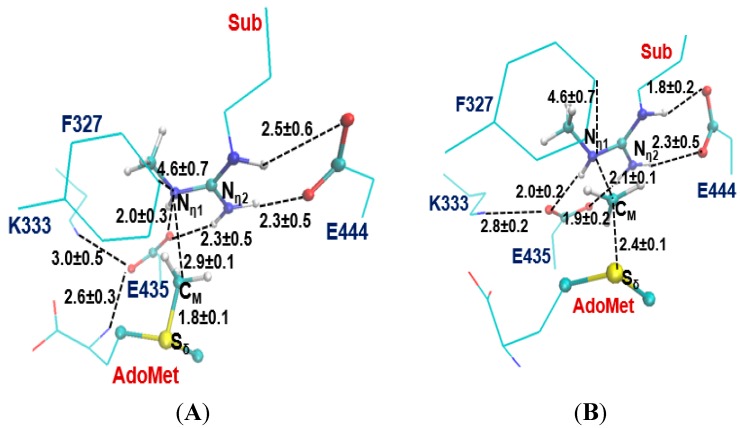
Results from the MD simulations for the 2nd methylation on N_η1_. (**A**) Active-site structure of the reactant complex; (**B**) Active-site structure near the transition state.

**Figure 8:**
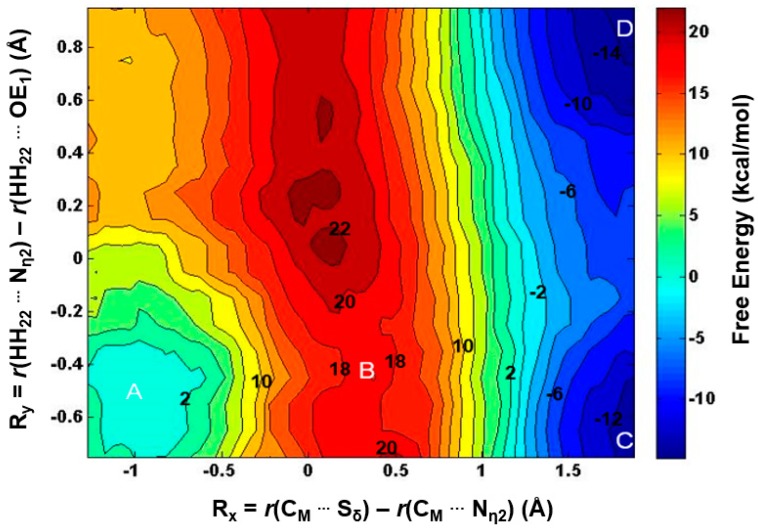
2D free-energy contour map for the 2nd methylation on N_η2_. Points A, B, and D designate the reactant, near transition state, and product complex, respectively. Point C corresponds to the hypothetical product complex with proton not being transferred, which is only 2 kcal/mol higher than Point D in the free-energy contour map. Some contour lines are shown with respective energy value in kcal/mol. The energy bar is shown on the right. The energy barrier is estimated to be around 18–19 kcal/mol.

The average structures of the reactant complex and near transition state for the 2nd methyl transfer to N_η2_ and N_η1_ are exhibited in Figure 6 and Figure 7, respectively. The active-site structures of the reactant complex (Figure 6A) show that the N_η2_ atom is well aligned with the methyl group of AdoMet. The relatively high barrier for the 2nd methyl transfer to N_η1_ appears due in part to the steric hindrance of F327 (as in the case of the 1st methyl transfer), although other factors may be involved as well. One interesting observation for the second methyl transfer is that the proton has not been transferred to the general base E435 at the transition state (see Figure 6B and Figure 7B). This is in contrast with the case of the first methyl transfer (shown in Figure 3B and Figure 4B) where the proton transfer occurs before the methylation reaches the transition state. This observation is also reflected in the 2D free energy map involving both the 2nd methyl transfer and the proton transfer (Figure 8). As is shown in the reaction path illustrated in Figure 8, the reaction reaches the transition state at point B without the deprotonation of the monomethylarginine.

Another interesting observation from Figure 6B and Figure 7B is that E435 forms a salt bridge to both N_η1_ and N_η2_ near the transition state. This salt bridge may be relatively strong compared to that in the reactant complex and help to stabilize the transition state; it may also play a role for the delayed proton transfer mentioned earlier. The free energy barrier is estimated to be 18–19 kcal/mol by the 2D free energy simulation (Figure 8), consistent with 20.1 kcal/mol from the 1D free energy simulations (Table 1).

## 3. Experimental Section

The simulation coordinates for the reactant complexes of methyl transfers were based on the crystallographic complex (PDB ID: 4GQB, 2.06 Å) of PRMT5 [23] that contains AdoMet analog and the H4 peptide with the methylation targeting substrate, Arg3. The coordinates of PRMT3 for comparison is from the crystal structure (PDB ID:1F3L, 2.06 Å) and modified based on the previous work [26]. The QM/MM MD and free energy (potential of mean force, PMF)) simulations were applied for monitoring the methylation processes and determining the free energy profiles with the CHARMM program [35]. A water sphere based on a modified TIP3P water model [36] with radius(r) of 30 Å, centered at CZ of Arg3, was pre-equilibrated to the system. A stochastic boundary with a Poisson-Boltzmann charge-scaling scheme [37] was applied for the model. The reservoir region had *r* > 22 Å, and the buffer region had r equal to 20 Å ≤ *r* ≤ 22 Å. The reaction region had *r* ≤ 20 Å. The -CH_2_-CH_2_-S^+^(Me)-CH_2_- part of AdoMet, the side chain of substrate Arg3/monomethylated Arg3, the side chains of E435 and E444 were treated by QM and the rest of the system by MM. The resulting systems contained around 5500 atoms with about 800 water molecules. The all-hydrogen potential function (PARAM27) [38] was used for the MM region, the self-consistence charge density functional tight binding (SCC-DFTB) [39,40] method was used for the QM region. The link-atom approach [41] was applied to separate the QM and MM regions.

The initial structures for the entire resulting system were optimized by use of the steepest descent (SD) and adopted-basis Newton-Raphson (ABNR) methods. The systems were gradually heated from 50 to 310.15 K in 50 ps. A 1 fs time step was applied to integrate equation of motion. 1.5 ns QM/MM MD simulations were initially executed for each of the reactant complex. The reaction coordinate was defined as a linear combination of *r*(C_M_^...^ S_δ_) and *r*(C_M_ ··· N_η1/2_), which is R = *r*(C_M_ ··· S_δ_) − *r*(C_M_ ··· N_η1/2_). The umbrella sampling method [42] in the CHARMM program along with the weighted histogram analysis method (WHAM) [43] was used to determine the free energy(PMF) change as a function of the reaction coordinate(s). The Monte Carlo Simulation bootstrapping integrated with WHAM [43] was applied 100 times to estimate the errors (shown in Figure 2 and indicated in the legend of Table 1) of the PMF profile. 20–22 simulation windows were saved for each methyl transfer process. And for each window 50 ps production runs were performed after 50 ps equilibration. The force constants of the harmonic biasing potentials used in the PMF simulations were 50–500 kcal·mol^−1^·Å^−2^. During the 50-ps production run of each window, structural coordinates were saved every 50 steps (1 fs/step), and consequently there are 1000 frames saved for each window. The distances shown in Figure 3, Figure 4, Figure 6 and Figure 7 are the means of corresponding distances with stand deviation (S.D.) collected from those 1000 frames. The 2D free energy (PMF) map were also determined with umbrella sampling method and two-dimensional-WHAM. The time step for simulations is 1 fs. The horizontal reaction coordinate for the 1st methylation on N_η1_, R_x_ = *r*(C_M_^...^ S_δ_) − *r*(C_M_^…^ N_η1_), was used to describe the methyl transfer process. And the vertical reaction coordinate, R_y_ = *r*(HH_12_^...^ N_η1_) − *r*(HH_12_^…^ OE_1_), was used to describe the proton transfer process. Similarly, for the 2nd methylations, the methyl transfer was explained by R_x_ = *r*(C_M_^...^ S_δ_) − *r*(C_M_^…^ N_η2_) and the proton transfer by R_y_ = *r*(HH_22_^...^ N_η2_) − *r*(HH_22_^…^ OE_1_). Approximated 300–400 windows were used in the construction of 2D free energy map, with 50 ps production run following 50 ps equilibration for each window. The force constants for each window were in the range of 100–800 kcal·mol^−1^·Å^−2^.

## 4. Conclusions

In this study, the Type II PRMT5 was investigated by the QM/MM MD and free energy simulations. E435 was found to function as a general base catalyst for accepting a proton from the substrate arginine during the 1st and the 2nd methylation. This residue seems to play a similar role as E144 in Type I PRMT3. The simulations provide the detailed mechanism for the symmetric di-methylation by the enzyme and suggest the possible role for some other key residues as well during the catalysis. The *k_cat_* for the arginine methylation catalyzed by PRMT5 is measured to be between 10 to 50 h^−1^, indicating that the activation energy barrier is about 18 kcal/mol based on transition state theory [23]. Thus, our estimates of the free energy barriers for the methyl transfers (19–20 and 18–19 kcal/mol for the first and the second methylation, respectively) are reasonable compared with the experimental observations. Furthermore, the simulations suggest that the symmetric di-methylation by PRMT5 seems to be energetically favorable, in agreement with the fact that PRMT5 is Type II PRMT. The proposed mechanism here based on the simulation results is different from the asymmetric dimethylation [25,26].
